# Dynamic Network Connectivity Reveals Markers of Response to Deep Brain Stimulation in Parkinson’s Disease

**DOI:** 10.3389/fnhum.2021.729677

**Published:** 2021-10-06

**Authors:** Chengyuan Wu, Caio Matias, Thomas Foltynie, Patricia Limousin, Ludvic Zrinzo, Harith Akram

**Affiliations:** ^1^Department of Neurological Surgery, Vickie and Jack Farber Institute for Neuroscience, Thomas Jefferson University, Philadelphia, PA, United States; ^2^Jefferson Integrated Magnetic Resonance Imaging Center, Department of Radiology, Thomas Jefferson University, Philadelphia, PA, United States; ^3^Unit of Functional Neurosurgery, UCL Institute of Neurology, London, United Kingdom; ^4^Victor Horsley Department of Neurosurgery, The National Hospital for Neurology and Neurosurgery, London, United Kingdom

**Keywords:** Parkinson’s disease, functional magnetic resonance imaging, dynamic functional connectivity, graph theory, deep brain stimulation

## Abstract

**Background:** Neuronal loss in Parkinson’s Disease (PD) leads to widespread neural network dysfunction. While graph theory allows for analysis of whole brain networks, patterns of functional connectivity (FC) associated with motor response to deep brain stimulation of the subthalamic nucleus (STN-DBS) have yet to be explored.

**Objective/Hypothesis:** To investigate the distributed network properties associated with STN-DBS in patients with advanced PD.

**Methods:** Eighteen patients underwent 3-Tesla resting state functional MRI (rs-fMRI) prior to STN-DBS. Improvement in UPDRS-III scores following STN-DBS were assessed 1 year after implantation. Independent component analysis (ICA) was applied to extract spatially independent components (ICs) from the rs-fMRI. FC between ICs was calculated across the entire time series and for dynamic brain states. Graph theory analysis was performed to investigate whole brain network topography in static and dynamic states.

**Results:** Dynamic analysis identified two unique brain states: a relative hypoconnected state and a relative hyperconnected state. Time spent in a state, dwell time, and number of transitions were not correlated with DBS response. There were no significant FC findings, but graph theory analysis demonstrated significant relationships with STN-DBS response only during the hypoconnected state – STN-DBS was negatively correlated with network assortativity.

**Conclusion:** Given the widespread effects of dopamine depletion in PD, analysis of whole brain networks is critical to our understanding of the pathophysiology of this disease. Only by leveraging graph theoretical analysis of dynamic FC were we able to isolate a hypoconnected brain state that contained distinct network properties associated with the clinical effects of STN-DBS.

## Introduction

Parkinson’s Disease (PD) is characterized by dopaminergic neuronal loss leading to cortico-basal ganglia-thalamo-cortical (CBGTC) dysfunction and the cardinal motor symptoms of resting tremor, rigidity, and bradykinesia (Hoehn and Yahr, 1967; Lees et al., 2009). The classical model of basal ganglia dysfunction has provided the framework for our understanding of PD pathophysiology and consequently shaped research in this field (Albin et al., 1989). Most human *in vivo* imaging studies have therefore focused on the CBGTC circuit. This includes resting-state functional MRI (rs-fMRI), which has the potential to identify basal neuronal activity and has been applied in PD to detect disease-specific changes (Wu et al., 2009, 2011; Helmich et al., 2010; Baudrexel et al., 2011; Hacker et al., 2012; Esposito et al., 2013; Szewczyk-Krolikowski et al., 2014; Akram et al., 2015; Hou et al., 2018; Ji et al., 2018).

Dopaminergic loss in PD has widespread effects that also result in numerous non-motor symptoms, which are closely interrelated with the readily observed motor symptoms (Chaudhuri et al., 2006; Schapira et al., 2017). This extensive involvement has been reported both in postmortem studies (Buddhala et al., 2015) and in advanced imaging studies of human subjects (Al-Bachari et al., 2014, 2017; Levin et al., 2014; Choi et al., 2016; Harrington et al., 2017; Nürnberger et al., 2017). Furthermore, imaging studies have supported the notion that the CBGTC circuit does not function in isolation, but rather, as a component of a more complex network involving diffuse regions throughout the brain (Wu et al., 2012, 2015; Hu et al., 2015; Müller-Oehring et al., 2015; Zhang et al., 2015, 2016; Koshimori et al., 2016; Mi et al., 2017; Tinaz et al., 2017; Gilat et al., 2018; Hou et al., 2018; Jia et al., 2019). It is therefore worthwhile considering possible interactions and influences from cortical regions that have not been implicated in classical models of PD pathophysiology.

Although previously unfeasible, such broad investigations are possible with graph theoretical analysis and have increased in popularity over the past decade. Graph theory allows for analysis of whole brain topography and identification of networks relevant to particular neurological diseases (Sporns, 2018; Farahani et al., 2019). In its application to rs-fMRI, each brain region of interest (ROI) serves as a *node* of the theoretical graph; and the resting state functional connectivity (FC) between two nodes serves as an *edge*. The *length* of a particular edge is the inverse of FC strength. Metrics based on edge lengths and patterns of FC can be used quantify local and global network organization throughout the brain (Wang, 2010).

Most rs-fMRI graph theory studies utilize static FC values derived from the entire rs-fMRI acquisition and as such, assume that a single functional brain state exists for the duration of the scan. It is more reasonable, however, that brain states are dynamic and change within seconds to minutes. Our hypothesis is that dynamic graph theory has greater potential to predict response to deep brain stimulation (DBS) as it may be more sensitive to changes occurring in specific brain states. We have previously characterized a distinctive pattern of basal ganglia FC associated with motor response to L-DOPA in advanced PD (Akram et al., 2017). Here, we extend this work to an analysis of distributed network properties using dynamic resting state functional connectivity (d-FC) that may be associated with DBS response. By using independent component graph theory analysis, we intended to interrogate global d-FC patterns associated with subthalamic nucleus deep brain stimulation (STN-DBS) response in patients with advanced PD. Ultimately, we aim to better understand the network characteristics that may predict therapeutic response.

## Materials and Methods

This study received ethical approval by the West London NHS Research Ethics Committee (10/H0706/68). All participants provided written informed consent.

### Study Subjects

Eighteen patients who met United Kingdom Brain Bank criteria for idiopathic PD underwent bilateral STN-DBS using an image-guided image-verified technique after selection by a multidisciplinary team of specialized movement disorders neurologists and functional neurosurgeons [Table 1].

**TABLE 1:** Patient information and response to treatments.

		Range	Mean ± SD
Age at implantation [years]		41– 70	55.3 ± 10.1
Sex	14 M, 4 F		
Hand dominance	18 RHD		
Predominant Symptom(s)			
Tremor	11 (61%)		
Rigidity	6 (33%)		
Bradykinesia	15 (83%)		
Dyskinesia	5 (28%)		
Dystonia	3 (17%)		
Disease duration [years]		4 – 22	11.1 ± 4.5
Hoehn and Yahr Stage		3 – 4	
Mini Mental Status Exam		28 – 30	29.6 ± 0.6
Preoperative UPDRS-III OFF		20 – 62	41.8 ± 11.3
Preoperative UPDRS-III ON		4 – 42	17.4 ± 10.1
Preoperative UPDRS-III L-DOPA Response [%]		32.3 – 91.5	60.5 ± 16.4
Preoperative L-DOPA equivalent daily dose [mg]		540 – 2160	1381.4 ± 447.4
Postoperative UPDRS-III ON-OFF		2 – 62	27.1 ± 16.8
Postoperative UPDRS-III ON-ON		1 – 34	12.8 ± 10.0
Postoperative UPDRS-III Improvement from STN-DBS [%]		19.2 – 81.3	53.5 ± 16.7
Postoperative L-DOPA equivalent daily dose [mg]		320 – 1266	747.8 ± 309.8

*Predominant symptoms listed are not mutually exclusive as patients may have more than one of these symptoms. L-DOPA equivalent daily doses are reported in milligrams. Clinical improvement in UPRDS-III is reported as a percentage change between assessments both performed at 1-year follow-up.*

All patients underwent neuropsychological screening with a Mini Mental Status Exam (MMSE) and a structural brain MRI to rule out dementia and significant brain atrophy, respectively. All patients’ UPDRS-III improved by at least a 30% preoperatively after administration of L-DOPA. For each patient, UPDRS-III scores were recorded postoperatively at 1-year follow-up in both the medication-ON and DBS-OFF (ON-OFF) and medication-ON and DBS-ON (ON-ON) states. The postoperative ON-OFF score was felt to also represent the state in which the initial rs-fMRI was acquired because improvement of UPDRS-III with L-DOPA postoperatively was correlated with preoperative L-DOPA response (*r* = 0.6527 [95%CI = 0.2211 0.8731], *p* = 0.008). Furthermore, use of the postoperative ON-OFF score accounts for disease progression and changes in response to L-DOPA over time. As such, the effects of STN-DBS over the 1-year follow-up were quantified as the percentage of improvement between the ON-OFF state and the ON-ON state.

### Magnetic Resonance Imaging Data Acquisition and Preprocessing

All patients were scanned preoperatively in the medication-ON state with a 3T-Siemens Magnetom-Trio MR-B17 with a well-padded 32-channel receive head coil. Image acquisition in the medication-ON state allowed for increased patient comfort and minimization of excessive involuntary head motion, which helps to decrease the likelihood of spurious findings in rs-fMRI (Tahmasian et al., 2015). Specifically, patients remained on their normal medication regimen and the MRI followed their morning medication dose. Although this interval varied between individual patients, motor symptoms were optimally controlled at the time of MRI acquisition for all subjects.

Multi-parameter mapping sequences were acquired for structural imaging. In brief, the whole protocol consisted of three 3D-FLASH acquisitions performed with T1, proton density (PD), and magnetization transfer (MT) weighting; these were paired with B1 (transmit field-mapping data to correct for the effect of inhomogeneous flip angles on the T1 maps) and B0 field map acquisitions (spatial resolution 1 × 1 × 1 mm^3^; repetition time 24.5 ms; multiple echo times; field of view 256 × 256mm; flip angle 6° [PD], 21° [T1], 6° [MT]; matrix size 256 × 256; partitions 176; total acquisition time of 26 min).

For rs-fMRI, patients were instructed to maintain a fixed gaze at a crosshair while multiecho echo planar imaging sequences were obtained in two successive acquisitions, for a total duration of 15 min (spatial resolution 3 × 3 × 2.5 mm^3^; repetition time 2.7 s; echo time 30 milliseconds; field of view 192 × 192 mm; flip angle 90°; 45 axial slices [2.5 mm thickness]; matrix size, 64 × 64; and a total of 512 scans).

### Preprocessing

Standard preprocessing of structural and functional MRI volumes was undertaken in the MATLAB-based CONN toolbox (version 19.c) (Whitfield-gabrieli and Nieto-castanon, 2012) to align images to an anatomical atlas and to reduce spatial and temporal artifacts. Structural scans were processed using the voxel-based quantification toolbox in SPM12 to generate MT and R1 maps (Weiskopf et al., 2013). The MT maps, which provide high contrast-to-noise ratio, were segmented to generate white matter, gray matter, and cerebrospinal fluid (CSF) maps using the “Segment” toolbox in SPM12 (Ashburner and Friston, 2005; Helms et al., 2009).

The first three volumes of each resting-state session were discarded. The remaining 253 functional volumes underwent realignment, unwarping, and slice-timing correction before undergoing rigid registration to the anatomical R1 scans and corresponding CSF and white-matter maps. Anatomical scans were then spatially normalized to Montreal Neurological Institute space (spatial resolution, 2 × 2 × 2 mm^3^); the resultant transformation was then applied to the functional data. Functional outlier detection was carried out using Artifact Detection Tools-based scrubbing^1^. The functional volumes were then inspected and smoothing was applied to reduce potential spatial and temporal artifacts using an 8-mm full-width at half-maximum Gaussian kernel.

To reduce spurious sources of variance in the functional data, denoising was carried out using the component-based noise correction method (Behzadi et al., 2007). The temporal time series with estimated subject motion (average framewise displacement calculated from 3 rotation and 3 translation parameters) and the BOLD time series within the subject-specific white matter and CSF masks, were used as temporal covariates and removed from the BOLD data using linear regression. Ultra-low-frequency fluctuations in the resulting residual BOLD time series were removed using a high-pass filter at 0.0078 Hz.

### Independent Component Extraction

**FIGURE 1:**
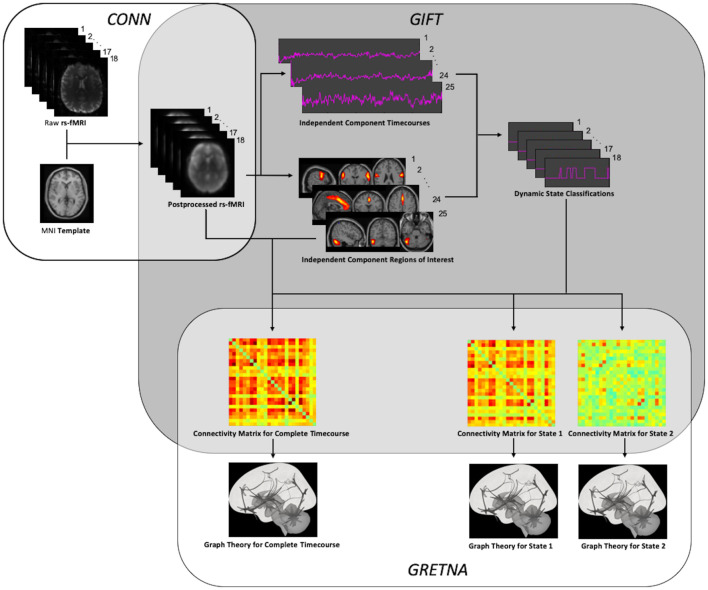
Processing Workflow. After preprocessing using SPM and the CONN toolbox, independent components were extracted to allow for both static and dynamic ICA using the GIFT toolbox. After thresholding and binarization of the resulting functional connectivity matrices, graph theory was performed using the GRETNA toolbox.

An overview of the entire processing workflow is illustrated in Figure 1. In order to take a data-driven approach that would not be biased by the application of a specific structural atlas, independent component analysis (ICA) was used to extract spatially independent components (ICs) from the rs-fMRI data. The Minimum Description Length (MDL) algorithm was first used to determine optimal number of components to be extracted from the preprocessed rs-fMRI data of each patient; and the mean number of components across the cohort was calculated. Group principal component analysis (PCA) were performed first on a subject-specific level and then on a group level to reduce rs-fMRI data to this mean optimal number of components. Spatial group ICA was then performed by applying the Infomax algorithm to find the ICs; and stability was assessed by running the algorithm 20 times in the ICASSO toolbox (Himberg and Hyvärinen, 2003). Group ICA back reconstruction was then performed to generate aggregated spatial maps of the ICs for the cohort. All steps for group ICA were performed using the GIFT toolbox (version 3.0b) (Calhoun et al., 2001).

Manual classification of ICs as signal or noise remains the gold standard and as such, the resultant components were inspected independently by two of the authors (CW and CM) to identify and eliminate noise components according to previously described classification methods (Griffanti et al., 2017). Discrepancies were handled by a consensus decision between the two reviewers. Based on spatial location, the remaining components were then grouped into one of eight well-described large-scale functional networks: salience network (Sa); default mode network (DMN); frontoparietal network (FPN); sensorimotor network (SM); cerebellar network (Ce); visual network (Vi); language network (La); or auditory network (Au).

### Static Resting State Functional Connectivity Analysis

Using the extracted ICs as ROIs, overall static FC was calculated for each subject. Only the BOLD signal within each subject-specific gray-matter mask was included in these calculations. Bivariate correlation coefficients were calculated for the entire BOLD time series between each ROI pair (*p* < 0.05) to generate the FC matrices for each patient. Multivariate analysis of covariance (MANCOVA) was then used to assess and incorporate covariates into the group representation of overall static FC (Allen et al., 2011). Given the known FC changes that occur with age and gender, (Allen et al., 2011; Ferreira et al., 2015; Kim et al., 2017) *age* at the time of DBS implantation and *gender* served as a control covariates. Since images were acquired during the medication-ON state the *preoperative UPDRS-III medication-ON score* was also incorporated as a control covariate in order to account for disease severity. While some studies have also used LEDD as a covariate, we do not believe this to be a reasonable strategy because of the variable bioavailability of oral dopamine replacement therapy, (Di Stefano et al., 2009; Tuite, 2016) which yields different therapeutic effects across patients. Instead, the UPDRS-III medication-ON score best represents the clinical state resulting from optimal medical therapy and therefore, the effects of L-DOPA on FC. Lastly, the *average framewise displacement* calculated during image preprocessing was also used as a control covariate in order to minimize the potential contribution of patient movement at the time of rs-fMRI acquisition on FC (Johnstone et al., 2006). In summary, age, gender, preoperative UPDRS-III medication-ON score, and subject motion during scan acquisition were incorporated as covariates in the calculation of the overall static FC matrix for the entire cohort. These steps for FC analysis were performed using the MANCOVAN toolbox within the GIFT toolbox.

### Static Resting State Graph Theory Analysis

In preparation for graph theory analysis, the resulting group FC matrix underwent thresholding to remove spurious connections and create a sparse graph. In order to analyze an efficient network capable of specialized, distributed, and integrated information processing, the small-world topology of a graph should be maximized with this thresholding step (Bassett and Bullmore, 2006; Langer et al., 2013). Rather than selecting an absolute threshold value, the graphs were binarized by maintaining a percentage of the strongest connections. Such proportional thresholding has been suggested to more favorable because it avoids the generation of graphs with different network densities across the study cohort, which in turn may bias the resulting calculation of network statistics (van Wijk et al., 2010). At the same time, proportional thresholding remains susceptible to bias from differences in overall functional connectivity between subjects. Therefore, for each patient, the overall functional connectivity was calculated as the mean of all positive values of the connectivity matrix so that it could be incorporated as a control covariate into the partial correlation analysis (van den Heuvel et al., 2017).

While the optimal proportional threshold is typically associated with the graph with maximal small-worldness, (Bassett and Bullmore, 2006; Langer et al., 2013) to accommodate for differences in optimal thresholds between graphs from different patients, we calculated the area under the curve (AUC) of each graph theory metric of interest over a range of thresholds (Koshimori et al., 2016; Kim et al., 2017). The small-worldness across this threshold range was also calculated to ensure that the graph with maximal small-worldness was included in this analytic range. For each metric, a total of 25 undirected and unweighted graphs were calculated by applying thresholds ranging from 10–34% at increments of 1% (Achard and Bullmore, 2007). The AUC was then calculated for each graph theory metric.

Although such properties have been previously described in detail, (Bullmore and Sporns, 2009; Bullmore and Bassett, 2011) it is instructive to familiarize ourselves with the metrics employed in this study. Global network properties were assessed with global efficiency and assortativity. *Global efficiency* represents the average strength of connection between all pairs of nodes in the network and as such, represents the extent of overall network integration (Medaglia, 2017). *Assortativity* indicates the degree to which nodes with similar degrees of connectivity are connected to each other (e.g., highly connected nodes connected to other highly connected nodes and poorly connected nodes connected to other poorly connected nodes) (Newman, 2002). Networks that are more assortative are considered to be more resilient – as removal or malfunction of a node has a lesser effect on the function of the overall network (Medaglia, 2017). Overlapping metrics of integration (e.g., *characteristic path length*) and resilience (e.g., *degree distribution*) were not calculated to avoid redundancy.

Local network properties were assessed with clustering coefficients and betweenness centralities for each ROI. The *clustering coefficient* is a measure of the proportion of a node’s neighbors that are also directly connected to each other. This metric therefore serves as an indication of the local network segregation. *Betweenness centrality* highlights the relative importance of a node in the communication efficiency of local network (Zuo et al., 2012). It is calculated by determining the number of shortest paths of a network that involve that particular node. As such, nodes with high values of betweenness centrality participate in a large number of shortest paths and have an important role in the information transfer within a network (Nigro et al., 2016). Overlapping metrics of segregation (e.g., *local efficiency*) and centrality (e.g., *closeness centrality*) were not calculated to avoid redundancy.

These four complementary graph metrics were each calculated across the aforementioned threshold range to calculate the AUC for each metric. All graph analysis calculations were performed using the GRETNA toolbox (v2.0.0) in the MatLab environment (Wang et al., 2015).

### Statistical Analysis of Static Functional Connectivity and Graph Theory Analysis

To assess the relationship between imaging characteristics and STN-DBS response, Pearson partial correlations were performed. Since the age, motion, and preoperative UPDRS-III ON score were already incorporated as control covariates in the calculation of the FC matrix, these variables were not incorporated into the partial correlations. For statistical analysis of the AUC of the graph theory metrics, *overall functional connectivity* was also used as a control covariate for the reasons delineated above. In order to correct for multiple comparisons, *p-*values underwent FDR correction when evaluating correlations for FC and local graph theory metrics. Statistical analysis was performed using the *RVAideMemoire* (version 0.9-77) and *stats* (version 3.6.3) packages within R Statistical Software (version 3.6.3; R Foundation for Statistical Computing, Vienna, Austria).

### Dynamic Resting State Functional Connectivity Analysis and Dynamic State Classification

Dynamic state classification was performed in a manner analogous to that described by the group from the University of Toronto using the GIFT toolbox (Kim et al., 2017; Díez-Cirarda et al., 2018). A sliding window of 22 TRs (59.4 s) with a 3 TR (8.1 s) Gaussian window and a step size of 1 TR (2.7 s) was applied to each subject’s rs-fMRI data, resulting in a total of 231 consecutive windows for dynamic analysis. For each window FC was calculated in a manner similar to that described for static FC; with the addition of L1 regularization to account for potential noise generated by the relatively short duration of the time windows. For each subject, 10 training repetitions were run in order to estimate the most appropriate regularization rate before application to the FC matrices.

Each of the 231 windows were then classified as a particular “brain state” based on the FC pattern by applying *k-*means clustering. To estimate the optimal number of brain states for classification, silhouette analysis was performed while varying the number of clusters between 2 and 10. The algorithm was run 100 times to reduce bias from random selection of cluster centroids. Bayesian information criterion (BIC) based on the Euclidean distance between clusters was then used to validate the result of the *k-*means clustering. In this manner, all 231 FC matrices for each subject could be appropriately classified into a particular brain state. The median FC values for all matrices classified as a particular brain state were calculated to generate a single d-FC matrix for that brain state. To better understand the significance of these states, the variance of FC across all subjects was also calculated; and compared to the variance in static FC. In addition, temporal properties of dwell times and number of state transitions were also calculated for further statistical analysis.

### Dynamic Resting State Graph Theory Analysis

For each d-FC matrix, graph metrics were calculated in the manner described above for static analysis. Once again, the AUC for the four graph theory metrics of interest were calculated with thresholds from 10–34%. It is again important to note that because small-worldness may be optimized at different thresholds for graphs across different subjects, this approach also allows for a uniform comparison of metrics across different graphs representing different brain states.

### Statistical Analysis of Dynamic Functional Connectivity and Graph Theory Analysis

Partial correlations were performed to assess the relationship between the temporal properties of d-FC (e.g., dwell times, number of state transitions) and clinical responses to STN-DBS. Analysis of d-FC itself and of the calculated graph metrics were performed for each state in the same manner described above for static analysis.

## Results

### Group Spatial ICA

**FIGURE 2:**
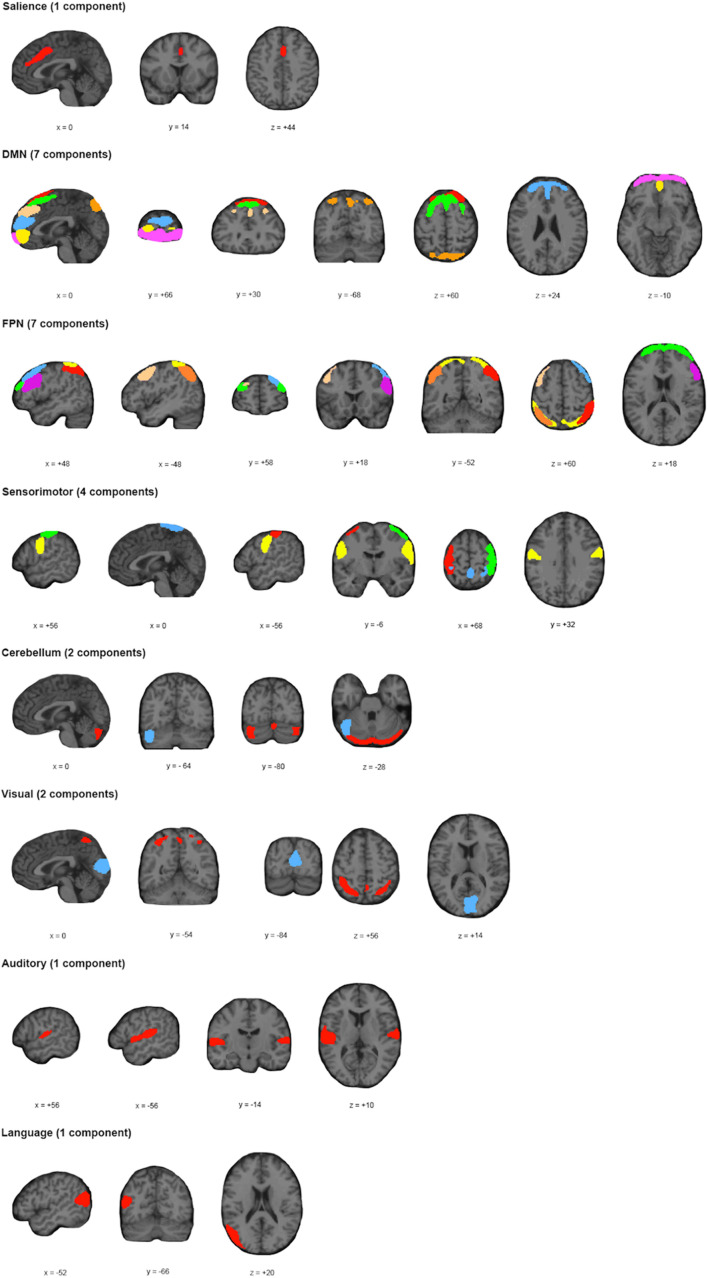
Spatial Location and Classification of Independent Components. A total of 25 independent components (ICs) were extracted from the dataset and subsequently classified into 8 known networks: salience network (Sa); default mode network (DMN); frontoparietal network (FPN); sensorimotor network (SM); cerebellar network (Ce); visual network (Vi); language network (La); or auditory network (Au). The color scheme allows for differentiation of components within a particular network; and does not represent any relationship with ICs of the same color for a different network.

Application of the MDL algorithm to the preprocessed rs-fMRI data yielded a mean optimal number of 66.579 ± 11.801 independent components. As such, data reduction with PCA reduced data first to 101 principal components based on subject-specific data, before arriving at 67 group independent components. After ICA and back reconstruction, the total number of ICs was reduced to 25 following manual classification. The 25 ICs were classified into 8 networks (Figure 2 and Table 2).

**TABLE 2:** Properties of Independent Components (ICs).

				MNI Peak Coordinates		
	’q	k	Tmax	X	Y	Z
Salience	0.945	7298	6.8	0	14	44
DMN	0.973	8042	12.8	0	60	18
	0.963	5074	17.6	−28	66	−6
	0.959	6273	14.1	10	−78	52
	0.934	8616	12.1	−26	62	2
	0.975	8889	11.9	38	62	−36
	0.978	4158	19	10	28	68
	0.977	8206	12.2	0	54	38
FPN	0.965	4324	16.5	−22	66	20
	0.971	5545	11.7	32	50	38
	0.969	5795	11.8	28	−64	64
	0.974	7746	12.2	−38	−62	56
	0.970	6636	11.4	48	−48	56
	0.961	7784	9.2	−50	14	40
	0.974	7236	13.8	56	18	28
Sensorimotor	0.972	6907	10.9	24	−40	76
	0.933	6603	10.1	58	−4	26
	0.974	6823	13.3	44	−36	64
	0.975	6529	13.6	−42	−38	64
Cerebellum	0.982	4607	15.6	−26	−90	−30
	0.952	6015	11	−48	−66	−28
Visual	0.897	9535	11.1	40	62	−36
	0.976	9608	8.8	−40	−50	56
Auditory	0.906	8833	10.9	−62	−18	10
Language	0.955	8759	9.4	−48	−76	22

*The stability/quality index (I_q_) over 20 ICASSO runs was high for all selected ICs. The maximum T score (T_max_) for each IC along with its location in MNI coordinate space are shown in the last two columns. IC masks were thresholded by μ + 4σ and minimum cluster size (k) ≥ 100.*

### Static Resting State MRI Analysis

**FIGURE 3:**
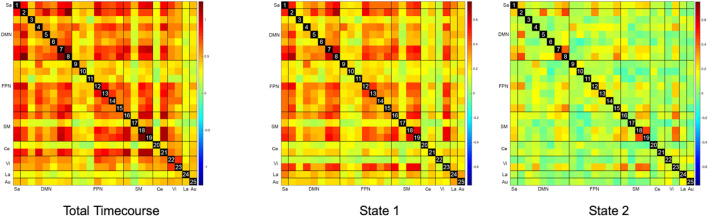
Static and Dynamic Functional Connectivity Matrices. The overall static resting state functional connectivity (Total Timecourse) of the whole brain shows a strongly interconnected network, which is also seen as a relatively hyperconnected state (State 1) in dynamic functional connectivity. A relatively hypoconnected state (State 2), which is actually the predominant brain state for most subjects was only revealed with dynamic functional connectivity analysis.

The overall static FC for the entire cohort is represented as the leftmost matrix in Figure 3. ROIs generally appear strongly interconnected – particularly ROIs within the DMN, SM, FPN, and Ce networks. There were no statistically significant correlations between FC and response to STN-DBS at one year after DBS implantation. Further analysis with graph theory metrics of global efficiency, assortativity, clustering coefficients, and betweenness centralities also failed to demonstrate any significant relationships with STN-DBS.

### Dynamic State Classification

Using both silhouette analysis and BIC for *k*-means clustering, categorization of d-FC into one of two brain states was determined to be optimal (Supplementary Figure 1).

Subjects generally spent more time overall in State 2 (67.03 ± 32.64%) than in State 1 (32.97 ± 32.64%); and also spent longer contiguous blocks of time in State 2 (4.62 ± 4.07 min) than in State 1 (1.00 ± 1.02 min). Six of the 18 patients spent more time in State 1 than in State 2. Across the cohort, patients transitioned between states an average of 4 ± 2.77 times. Time spent in a particular state, dwell time, and number of transitions were not significantly correlated with response to STN-DBS.

### Dynamic Resting State MRI Analysis

The representative FC matrices for the entire cohort for each brain state are illustrated on the right-hand panels of Figure 3. While the d-FC State 1 appears similar to the overall static FC and relatively hyperconnected, State 2 represents a relatively hypoconnected state, with stronger connections only remaining in portions of the SM, DMN, and Vi networks.

**FIGURE 4:**
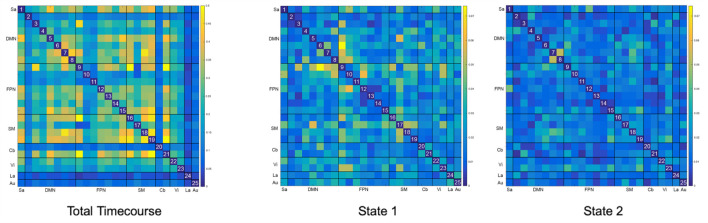
Between-Subject Variance for Static and Dynamic Functional Connectivity Matrices. The variance seen with overall static resting state functional connectivity is an order of magnitude higher (μ = 0.204) than the variance seen with both dynamic resting states (μ = 0.022 and μ = 0.016). Please note that the color scale for the total timecourse (0–0.5) is different than for the dynamic states (0–0.75) in order to accommodate this significant difference.

Of note, variance in FC across subjects decreased by an order of magnitude for both d-FC states (Figure 4). While mean variance for the total timecourse was 0.204 (range 0 – 0.541), mean variance for State 1 was 0.022 (range 0 – 0.074) and mean variance for State 2 was 0.016 (range 0 – 0.053).

**FIGURE 5:**
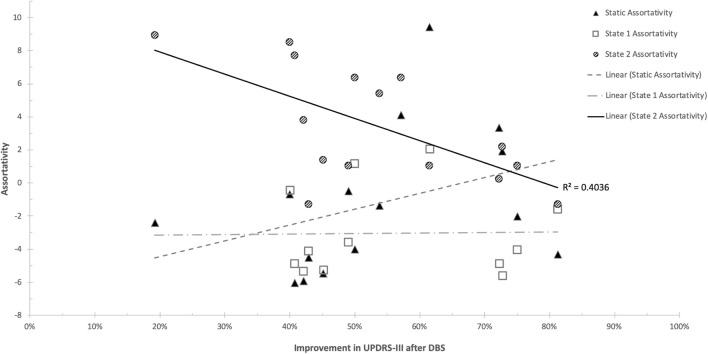
Scatterplot of Network Assortativity and UPDRS-III improvement. Significant findings were present only in the hypoconnected state (State 2).

There were no statistically significant correlations between d-FC measures and response to STN-DBS at one year after DBS implantation. Further analysis with graph theory metrics demonstrated significant relationships with STN-DBS only in the hypoconnected state (State 2). Specifically, response to STN-DBS was negatively correlated with network assortativity (*r* = −0.6907 [95%CI = −0.8937 −0.2528], *p* = 0.006) (Figure 5). No other graph metrics were significantly correlated with STN-DBS.

## Discussion

In this study, we identified a whole brain network characteristic that may predict response to STN-DBS for advanced PD. To date, a number of studies have used graph theory of whole brain rs-fMRI to quantify large scale network changes seen in early to mid-stage PD. Despite global FC changes, small-world order and overall organization remain grossly intact (Luo et al., 2015; Ma Q. et al., 2017; Suo et al., 2017). Meanwhile, a breakdown of local network integrity occurs with increased segregation and disconnection of subnetworks, which lack normal integration with one another (Luo et al., 2015; Tinaz et al., 2017; Vancea et al., 2019). Specifically, there is a reliable decrease in sensorimotor integration – explaining the predominant manifestation of motor symptoms in PD (Wu et al., 2009; Luo et al., 2015; Koshimori et al., 2016; Ma L. Y. et al., 2017; Ma Q. et al., 2017; Suo et al., 2017). Furthermore, disruption of network integration has been identified in associative regions including prefrontal and parietal cortices (Wu et al., 2009; Koshimori et al., 2016; Ma Q. et al., 2017; Tinaz et al., 2017; Vancea et al., 2019). Lending more credence to these early findings, the magnitude of network disruption has not only been associated with disease severity, (Wu et al., 2009; Zhang et al., 2015; Gu et al., 2017; Suo et al., 2017) but also been shown to improve with L-DOPA (Wu et al., 2009; Berman et al., 2016; Vancea et al., 2019).

While these studies have relied on methods that average the entire time course of the rs-fMRI signal and characterize a single functional brain state, it is more plausible that multiple brain states exist and change throughout the duration of the acquisition. Dynamic FC (d-FC) analysis of rs-fMRI in healthy subjects has been used to quantify these temporal changes in FC, which are thought to represent the dynamic coordination between different functional networks (Chang and Glover, 2010; Kang et al., 2011; Allen et al., 2014; Calhoun Vince et al., 2014). More recently, d-FC has been used to identify potential biomarkers of neurological diseases – including Parkinson’s Disease (Kim et al., 2017; Díez-Cirarda et al., 2018; Fiorenzato et al., 2019; Navalpotro-Gomez et al., 2020). Of note, these studies have also employed graph theory to facilitate interpretation of these dynamic whole-brain network effects.

We therefore leveraged d-FC to account for potential differences in FC that exist between different brain states. Such differences are highlighted by the significant decrease in between-subject variance seen with d-FC relative to static FC in this study. We also used a data-driven approach with ICA to overcome limitations associated with the use of standardized anatomical atlases, which can significantly alter the results of FC analysis. Of note, the functional brain states we identified are comparable to results of previous studies using d-FC analysis to characterize patients with PD (Kim et al., 2017; Díez-Cirarda et al., 2018; Fiorenzato et al., 2019; Navalpotro-Gomez et al., 2020). As with those studies, we isolated a hypoconnected state, in which subjects spent the majority of time; as well as a hyperconnected state, which occurred less frequently. Unfortunately, the interpretation of the physiologic role of these two states remains unclear – while some studies have associated the hyperconnected state with PD and disease severity, (Kim et al., 2017; Díez-Cirarda et al., 2018) others present conflicting findings with the hypoconnected state being more prevalent in PD (Fiorenzato et al., 2019; Navalpotro-Gomez et al., 2020). Since both states have been implicated as a potential pathological state, it is unlikely that simply represent overall PD severity. It is possible, however, that these states reflect different PD subtypes, as work associating specific dynamic resting states with motor and non-motor symptoms remains limited.

In our data-driven approach, we only identified a significant rs-fMRI property in the hypoconnected state. While we did not find any significant relationships between static FC measures or static graph theory metrics and STN-DBS response, d-FC allowed us to identify that network assortativity was strongly correlated with STN-DBS response only in this hypoconnected state. Even though the hypoconnected state predominates the rs-fMRI acquisition, the hyperconnected state appears to significantly impact static whole brain topography – obscuring FC relationships in the hypoconnected state. As a result, analysis of static FC alone may prevent the identification of significant findings that only exist in a particular state. We have therefore highlighted the potential of dynamic graph theoretical analysis to identify specific network connectivity patterns that would otherwise be missed with other approaches.

As we interpret these findings, it is important to remember that rs-fMRI in this study was obtained during the medication-ON state for purposes of patient comfort, to minimize of excessive involuntary head motion, and for ethical reasons. Several studies have taken this same approach to further our understanding of functional networks in PD with the recognition that the network properties reflect a combination of the disease itself and its response to medical treatment (Tahmasian et al., 2015).

As a measure of network resilience, assortativity may be interpreted as the ability of the brain to efficiently transfer information throughout the entire network. While assortativity has been found to decline in multiple sclerosis and neurodegenerative diseases; (Bahrami and Hossem-Zadeh, 2015; Llufriu et al., 2019) it has also been noted to be elevated in pathologies with hyperexcitable states, such as epilepsy and chronic insomnia (Kinjo et al., 2018; Li et al., 2018; Lim et al., 2019). While use of network assortativity has been limited in PD, one study reported a positive correlation between assortativity and cognitive performance in PD (Lin et al., 2018). In this study, we found that lower baseline network assortativity, representing deterioration of network resilience and information transfer, was strongly correlated with greater motor improvement with STN-DBS. Given our study design, it remains unclear if this finding represents a network pattern characteristic of PD or if it represents network changes induced by L-DOPA. Regardless, the association between whole brain network assortativity and motor response to STN-DBS is clear.

### Limitations

For patient comfort and to reduce motion artifact, all patients were on optimal L-DOPA medication at the time of the preoperative MRI scan. As such, we were not able to directly assess the effects of L-DOPA and DBS on BOLD signal. At the same time, since our aim was to evaluate the potential of rs-fMRI to predict treatment to DBS, we only used data that could be reasonably obtained only prior to surgery.

While the relatively small sample size may be considered a limitation of this work, the 18 subjects enrolled in the current study is comparable to other rs-fMRI studies involving PD patients (Tahmasian et al., 2015). Furthermore, despite this potential limitation, the identified correlations are strong and certainly warrant further investigation. Another limitation of this work is the relative heterogeneity of the study population. Since there exists no standard diagnostic criteria for characterizing advanced PD, (Fasano et al., 2019) there still exists variability in terms of age, disease severity, and motor outcomes. Nevertheless, we did control for such factors in an attempt to perform the most robust analysis possible with the existing data. Ultimately, we believe that the heterogeneity of our cohort benefits this study, as the results are more generalizable to the population of interest – namely those that may be potential candidates for DBS.

It is also important to note that no ICs were identified within the basal ganglia. While the reason for this deficiency remains unclear, the data-driven approach we have taken in our analysis prevents us from forcing the inclusion of this region. Even upon careful review of the 67 original ICs, there was no IC with meaningful signal within the basal ganglia. We must therefore keep this in mind in the interpretation of our results, as the importance of the basal ganglia cannot be refuted in the study of PD. In the current study, we are unable to make any conclusions about their role in predicting STN-DBS response.

The lack of basal ganglia ICs is one reason we are unable to make direct comparisons with our prior work on L-DOPA response in an overlapping cohort of patients (Akram et al., 2017). Given the overlapping cohort used in the present study and our prior work on FC in predicting L-DOPA response, it would be useful to draw comparisons between these two analyses – particularly given the known relationship between L-DOPA and DBS response. Significant differences in methodology, however, also prevent us from making such comparisons. Our prior work, employed a hypothesis-driven approach in which a narrow scope of analysis was performed solely on predefined regions of interest defined on the FSL Harvard-Oxford cortical structural atlas, the ATAG subthalamic nucleus atlas, and the ATAG MNI04 BG atlas. Static functional connectivity was then analyzed to investigate relationships between FC and L-DOPA response. The current study, however, takes a data-driven approach using ICA-defined ROIs, which generates different regions for analysis than with our prior work. Furthermore, instead of seed-to-seed analysis in which individual connections are being analyzed, we have taken a whole-brain network approach with graph theory analysis. We have also employed dynamic resting-state analysis to further the sensitivity of the analysis by incorporating the investigation of different brain states. With that said, if independent components had been identified within the basal ganglia, we could potentially have made direct comparisons with this prior work.

Given the strict criteria implemented for statistical significance, it is certainly possible that there are other relevant relationships that have been overlooked due to the sample size of our cohort and type II error. Future endeavors to replicate this study or with larger cohorts capable of providing more statistical power will provide a better understanding of these relationships.

## Conclusion

We submit that distinct dynamic network properties may play a significant role in response to STN-DBS in PD. Given the widespread effects of dopamine depletion in PD, analysis of whole brain networks is critical to understanding its pathophysiology. We leveraged graph theoretical analysis of dynamic functional connectivity to characterize local and global network organization in patients with advanced PD who underwent STN-DBS. Only by separating functional brain states were we able to identify distinct network properties associated with STN-DBS. Specifically, motoric improvement with DBS was associated with decreased network resilience. Further studies are required to elucidate these possible relationships.

## Data Availability Statement

Since data for the current analysis was acquired from patients undergoing routine care, institutional regulations require the establishment of formal data sharing agreements before patient identifying information included in this dataset or metadata can be shared. Deidentified raw data can be provided upon request to the corresponding author, after approval from the institutional review board.

## Ethics Statement

The studies involving human participants were reviewed and approved by West London NHS Research Ethics Committee. The patients/participants provided their written informed consent to participate in this study.

## Author Contributions

CW did the conceptualization, methodology, software, validation, formal analysis, investigation and writing – original draft, and visualization. CM did the methodology, validation, formal analysis and writing – review and editing. TF, PL, LZ did the resources, data curation, and writing – review and editing. HA did the resources, data Curation, formal analysis, writing – review and editing, and supervision. All authors contributed to the article and approved the submitted version.

## Conflict of Interest

The authors declare that the research was conducted in the absence of any commercial or financial relationships that could be construed as a potential conflict of interest.

## Publisher’s Note

All claims expressed in this article are solely those of the authors and do not necessarily represent those of their affiliated organizations, or those of the publisher, the editors and the reviewers. Any product that may be evaluated in this article, or claim that may be made by its manufacturer, is not guaranteed or endorsed by the publisher.

## Footnotes

1
www.nitrc.org/projects/artifact_detect/

## Supplementary Material

Supplementary Figure 1 Determination of Optimal Number of Clusters for Dynamic Resting State Functional Connectivity Analysis. Using silhouette analysis, the maximal value was found to result from 2 clusters. Upon validation with Bayesian information criterion (BIC) based on the Euclidean distance between clusters, it was found that the data would be optimally grouped into either 2 or 3 clusters. As such, it was determined that each window of the dynamic resting state analysis would be classified into one of two brain states.

## Abbreviations

Au: auditory network
AUC: area under the curve
BA 7: Brodmann area 7
BIC: Bayesian information criterion
Ce: cerebellar network
CBGTC: cortico-basal ganglia-thalamo-cortical
CSF: cerebrospinal fluid
DBS: deep brain stimulation
DMN: default mode network
FPN: frontoparietal network
ICA: independent component analysis
ICs: independent components
La: language network
MANCOVA: multivariate analysis of covariance
MDL: minimum description length
MMSE: mini mental status exam
PCA: principal component analysis
ROI: region of interest
rs-fMRI: resting-state functional MRI
FC: resting state functional connectivity
d-FC: dynamic resting state functional connectivity
Sa: salience network
SM: sensorimotor network
STN: subthalamic nucleus
Vi: visual network.
