# Supplementary material for: Dynamic Network Connectivity Reveals Markers of Response to Deep Brain Stimulation in Parkinson’s Disease
Source: Front Hum Neurosci. 2021 Oct 6;15:729677. doi: 10.3389/fnhum.2021.729677 (PMC8526554; doi:10.3389/fnhum.2021.729677)
Supplement: Supplementary Figure 1 — Determination of Optimal Number of Clusters for Dynamic Resting State Functional Connectivity Analysis. Using silhouette analysis, the maximal value was found to result from 2 clusters. Upon validation with Bayesian information criterion (BIC) based on the Euclidean distance between clusters, it was found that the data would be optimally grouped into either 2 or 3 clusters. As such, it was determined that each window of the dynamic resting state analysis would be classified into one of two brain states. [file Data_Sheet_1.PDF]

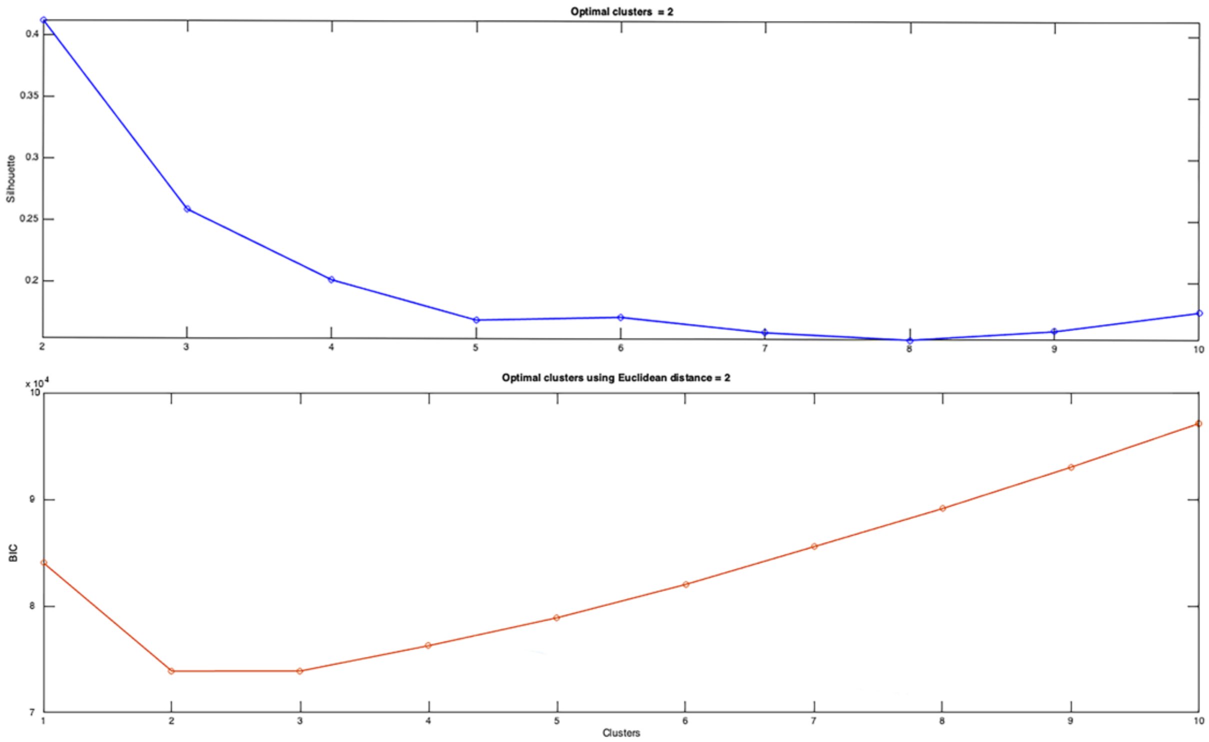

Supplementary Figure 1 – *Determination of Optimal Number of Clusters for Dynamic Resting State Functional Connectivity Analysis*. Using silhouette analysis, the maximal value was found to result from 2 clusters. Upon validation with Bayesian information criterion (BIC) based on the Euclidean distance between clusters, it was found that the data would be optimally grouped into either 2 or 3 clusters. As such, it was determined that each window of the dynamic resting state analysis would be classified into one of two brain states.
